# Serum insulin-like growth factor-1 and leptin concentrations in Black Bengal goats at different ages and gestation periods and their relationship to kid growth performance

**DOI:** 10.14202/vetworld.2024.1397-1404

**Published:** 2024-06-28

**Authors:** Chollada Buranakarl, Sumonwan Chamsuwan, Morakot Nuntapaitoon

**Affiliations:** 1 Department of Physiology, Faculty of Veterinary Science, Chulalongkorn University, Bangkok, 10330, Thailand; 2 Department of Obstetrics, Gynaecology and Reproduction, Faculty of Veterinary Science, Chulalongkorn University, Bangkok, 10330, Thailand; 3 Multi-Omics for Functional Products in Food, Cosmetics and Animals Research Unit, Chulalongkorn University, Bangkok, 10330, Thailand

**Keywords:** Black Bengal goat, growth performance, insulin-like growth factor 1, leptin

## Abstract

**Background and Aims:**

The impact of maternal hormone concentration on kid growth performance in relation to insulin-like growth factor 1 (IGF-1) and leptin is minimal. This study examined IGF-1 and leptin levels at varying ages and gestation periods for their correlation with Black Bengal goat kids’ growth during the preweaning phase.

**Materials and Methods:**

Blood samples were collected from 43 dams with different reproductive cycles and 28 prepubertal goats to measure serum concentrations of IGF-1 and leptin. Among dams, both hormones were investigated in different age ranges (<2, 2–3, 3–4, and >4 years old) and reproductive cycles (non-pregnancy, early gestation (1–50 days), mid-gestation (51–100 days), late gestation (101–135 days), and the last 15 days before delivery). After delivery, 65 kids from 34 dams were weighted weekly for 8 weeks to calculate average daily weight gain (ADG) at 0–4 weeks (ADG0–4 W) and 4–8 weeks (ADG4–8 W) and growth performance, including weight (W), height (H), length (L), chest girth (C) measured at birth (W0, H0, L0, and C0) and at 10 weeks of age (W10, H10, L10, and C10) were related to hormone serum concentrations in their dams at different gestation periods including the last 15 days before delivery.

**Results:**

Dams had higher mean serum IGF-1 (p < 0.001) and leptin (p < 0.05) than prepubertal goats. Dams at late gestation had higher IGF-1 concentrations than those at early and mid-gestation and during the last 15 days before delivery. However, it was consistent with non-pregnant goats. The kid’s growth performance correlated positively with IGF-1 concentration, which was collected in the last 15 days before delivery. Multivariate analysis showed that ADG0–4 W was higher in kids born from dams with high IGF-1 than those with low IGF-1 measured during the last 15 days of delivery, whereas leptin tended to have a similar effect.

**Conclusion:**

Serum IGF-1 and leptin concentrations of dams measured during the last 15 days before delivery were associated with kid’s growth during the preweaning period.

## Introduction

Black Bengal (BB) goats are known as meat-type small to medium-sized goats that produce many kids in one litter. They have been raised in subtropical areas, especially in Asia, because of their tolerance to hot environments, disease resistance, low feed demand, and high quality of meat and skin [1]. Like other farm animals, the growth performance of preweaning kids is one of the most interesting areas, which can be influenced by many factors such as genetics, nutritional intake, and environment. Measuring body weight and conformation during pre-weaning is essential for genetic selection and farm management monitoring to achieve high economic values.

During the pre-weaning period, a kid’s growth can be determined by many factors, such as genetics, farm management, colostrum, feed intake, litter size, and disease status. The neuroendocrine system, such as the somatotrophic axis, is one of the most important factors that regulate intake and nutrient use. Insulin-like growth factor-1 (IGF-1) is a hormone that can regulate growth, especially during early development. After parturition, IGF-1 is passively transferred through colostrum, which has a very high concentration and declines dramatically to a very low concentration a few days after birth [2]. IGF-1 is essential for gastrointestinal development and enhances early neonatal growth and immunity [3]. The concentration of IGF-1 in colostrum was associated with the pre-weaning growth rate [4]. Moreover, a positive correlation was demonstrated between plasma IGF-1 levels and body trait measurements in prepubertal male and female White and Angora goats [5]. Leptin is another crucial hormone in regulating appetite, energy expenditure and balance, growth, reproduction, body composition, and immunity [6]. High leptin concentration inhibits feed intake by binding to hypothalamus receptors and regulating fat distribution and storage. Plasma leptin concentration increases with increasing body fat. Leptin expression was associated with requirements under many physiological states, such as food intake, growth, and reproductive cycle. Unlike IGF-1, there is no evidence of leptin accumulation in the colostrum prepartum [7]. However, higher expression of leptin mRNA and protein in the gastrointestinal tract of calves than in mature cows has been demonstrated [8], while the role of leptin during a pre-weaning period is still unclear.

Due to the limited information regarding age and gestation period in relation to circulating IGF-1 and leptin concentrations and their relationship with pre-weaning growth performance of kids, the objectives of this study were to investigate the circulating concentrations of IGF-1 and leptin in immature goats and in dams with different gestation periods and determine whether their concentrations from different gestation periods are associated with pre-weaning growth performance of kids.

## Materials and Methods

### Ethical approval

The protocol was approved by the Institutional Animal Care and Use Committee, Faculty of Veterinary Science, Chulalongkorn University (protocol number 2231055).

### Study period and location

The experiment was conducted from November 2022 to October 2023 at the Chaipattana Foundation’s Black Bengal Goat Domestication Project in Chiang Rai province in Northern Thailand at 20º18’ 42.4’ N 99º49’ 06.5’ E.

### Animal, housing, and management

The prepubertal goats contained 16 females and 12 males with an average age of 7.5 ± 0.1 (range 7.1–8.7) months. Among 43 dams, the average age of dams at blood collection was 2.78 ± 0.18 (range 0.85–5.69) years, whereas the average parity number was 2.47 ± 0.24 (range 1–6). There were 19, 7, 4, 8, 4, and 1 dams with parity numbers of 1–6, respectively. The number of dams aged <2, 2–3, 3–4, and > 4 years was 15, 10, 10, and 8, respectively. The gestation period was classified as non-pregnancy (n = 6), early gestation (1–50 days) (n = 9), mid-gestation (51–100 days) (n = 9), late gestation (101–135 days) (n = 12), and 15 days before delivery (136–150 days) (n = 7). To study the relationships of dam’s IGF-1 and leptin on kid’s growth performance, 34 dams along with 27 female and 38 male offspring from the next parity were recruited. Nine dams were excluded due to non-pregnant (n = 6) and abortion during the last trimester (n = 3).

All goats were housed in a conventional open housing system with 2–3 goats/pen (3 × 3 meters). Food was divided and given twice a day at approximately 7:00 h and 18:00 h while all goats had access to free water. The diet consisted of concentrate giving approximately 2% of body weight (91.2% dry matter [DM] and chemical composition of 17.2% crude protein, 2.92% fat, 12.6% crude fiber, 14.3% ash, 34.1% neutral detergent fiber [NDF], and 16.4% acid detergent fiber [ADF] per DM basis). The roughage was also given by approximately 3 kg/head/day (w/w) of chopped fresh Napier grass (16.2% DM with 15.0% crude protein, 1.85% fat, 30.76% crude fiber, 11.3% ash, 61.2% NDF, and 39.3% ADF per DM basis) and Pangola hay (92.8% DM with 3.54% crude protein, 1.21% fat, 30.59% crude fiber, 5.42 ash, 66.2% NDF, and 37.6% ADF per DM basis) at a ratio of 2:1. All goats were allowed to walk without grazing in the field between 9:00 and 12:00 h.

Goats were mated and delivered naturally. The pregnant dam was transferred to an individual pen 1 week before delivery. After parturition, newborn kids stayed with their mothers and received colostrum and milk until weaning at approximately 13 weeks. The kids were allowed to consume the dam’s food, both concentrate and roughage, in the same pen, which would be started around 1 month of age. However, kids born from high litter-size dams that produced inadequate colostrum may receive milk replacers and be excluded from this study. All animals received routine health checks daily and were vaccinated against the foot-and-mouth disease virus. Blood test monitoring for negative results of caprine arthritis encephalitis virus and brucellosis was routinely performed every year. Anthelmintic medication was applied periodically to eradicate both gastrointestinal parasites and ectoparasites.

### Blood sampling

Blood samples for IGF-1 and leptin concentrations were taken from the jugular vein at 10:00 h in all 43 dams and 28 prepubertal goats on the same day. Approximately 5 mL of blood was collected into a plain tube without anticoagulant (Monovette®, Numbrecht, Germany), immediately placed on ice, and centrifuged at 2,500× *g* for 5 min. The serum was separated and stored at −70°C for later analysis of IGF-1 and leptin concentrations.

### Determination of serum IGF-1 and leptin levels

The concentration of IGF-1 was determined using electrochemiluminescence techniques for immunoassay (Cobas e801, Roche Diagnostics, Basel, Switzerland). Leptin was measured using a goat-specific leptin commercial sandwich enzyme-linked immunosorbent assay kit (CUSABIO, catalog number: CSB-E12834G, Biotech Co., Ltd, China) according to the manufacturer’s instructions. The sensitivity was <0.156 ng/mL and its detection range was 0.625–40 ng/mL. The intra- and inter-assay coefficients of variation were <8% and <10%, respectively.

### Measurement of body conformation

All information regarding the identity, age, sex, litter size, parity, and date of birth of all goats were recorded and obtained from the farm. The information on the conformation and weight of the kids was collected when they were born between December 2022 and July 2023. Neonates were routinely separated from their mothers within 3 h after parturition to measure conformation at birth, including birth weight, length (L0), height at the withers (H0), and chest girth (C0), using tape. The weight was recorded while they were placed inside the basket over an electronic digital balance (CDR-30, CST Instruments, Taiwan). Body length was measured from the shoulder point to the pin bone. Height was measured from the ground at the front hoof to the point of the withers. Chest girth was measured as the body circumference at the heart, just behind the elbows, and up to the withers. All conformations were remeasured at 10 weeks postpartum (W10, L10, H10, and C10). Body weight was measured weekly after birth until 8 weeks of age, and the average daily gain (ADG) was calculated during weeks 0–4 (ADG0–4 W) and weeks 4–8 (ADG4–8 W) postpartum.

### Statistical analysis

All analyses were performed using Statistical Analysis System (SAS) version 9.4 (SAS Institute Inc., USA). Data are presented as mean ± standard error of mean. IGF-1 and leptin concentrations of dams and prepubertal goats and sex differences were analyzed using the general linear model (GLM) procedure of SAS, while sex was excluded from the model because of non-significant differences. The age of dams was classified into four classes: <2 (n = 15), 2–3 (n = 10), 3–4 (n = 10), and more than 4 years old (n = 8). The gestation period was classified as non-pregnancy (n = 6), early gestation (1–50 days) (n = 9), mid-gestation (51–100 days) (n = 9), late gestation (101–135 days) (n = 12), and the last 15 days before delivery (n = 7). Serum IGF-1 and leptin concentrations at different ages and gestation periods were compared using the GLM of SAS. Relationships among age, gestation period, IGF-1 and leptin concentrations, and kid growth were determined using Pearson correlation. The relationships between serum IGF-1 and leptin concentrations of dams during early, mid-, and late gestation and the last 15 days before delivery were evaluated with growth performance from 65 offspring. The kid’s growth performances (i.e., weight, length, height, and chest) at days 0 and week 10 after birth and ADG between weeks 0–4 (ADG0–4 W) and 4–8 (ADG4–8 W) after birth were analyzed using a general linear mixed model procedure (MIXED) of SAS. Kid’s growth performance was regarded as the dependent variable in each model. The IGF-1 and leptin concentrations in the dams were classified into two classes based on the average mean values (IGF-1; low: ≤231.6 ng/mL and high: >231.6 ng/mL, or leptin; low: ≤3.0 mg/dL and high: >3.0 mg/dL). The final model included the concentration of IGF-1 or leptin classes as fixed effects and the kid litter size as covariates. The dam identification number was added to the statistical model as a random effect. The least-square means were obtained from statistical models. Values with p < 0.05 were considered statistically significant, while 0.10 > p > 0.05 was considered a tendency.

## Results

### Serum IGF-1 and leptin levels in dams and prepubertal goats

The IGF-1 and leptin concentrations of the dams and prepubertal goats are shown in Table-1. The dam had significantly higher serum concentrations than prepubertal goats for both IGF-1 (p < 0.001) and leptin (p < 0.05).

**Table-1:** Serum IGF-1 and leptin concentrations in dams and prepubertal goats.

Groups	IGF-1 (ng/mL)	Leptin (ng/mL)
Prepubertal goat (28)	126.5 ± 18.6	0.56 ± 0.74
Dams (43)	291.8 ± 15.0***	2.92 ± 0.61*

Data are presented as mean ± SEM.

* p < 0.05,

*** p < 0.001. Number in parentheses indicates the number of animals. IGF-1=Insulin-like growth factor 1

### Serum IGF-1 and leptin concentrations in dams with different age and gestation period

Among the 43 dams, the age of the dams was not correlated with IGF-1 and leptin concentrations. No difference in IGF-1 and leptin concentrations was found among age groups (Table-2).

**Table-2:** IGF-1 and leptin levels in dams with different ages and gestation periods.

Parameter	Number	IGF-1 (ng/mL)	Leptin (ng/mL)
Age (years)			
<2	15	273.8 ± 27.8	2.26 ± 1.37
2–3	10	310.1 ± 34.2	1.18 ± 1.68
3–4	10	302.6 ± 34.5	6.00 ± 1.79
>4	8	258.3 ± 37.6	2.18 ± 1.93
Gestation periods			
Non-pregnancy	6	323.6 ± 43.8^ab^	1.27 ± 2.15
Early gestation (1–50 days)	9	178.9 ± 36.6^c^	5.00 ± 1.79
Mid-gestation (51–100 days)	9	284.4 ± 36.3^b^	1.57 ± 1.78
Late gestation (101–135 days)	12	386.4 ± 29.6^a^	3.41 ± 1.45
The last 15 days before delivery (136–150 days)	7	257.8 ± 38.7^bc^	3.27 ± 2.21

Data are presented as mean ± SEM, number in parenthesis indicates gestation length. Different superscripts (a, b, c) indicate statistical significance (p < 0.05). IGF-1=Insulin-like growth factor 1

A significant positive correlation was found among pregnant dams between IGF-1 and the day of gestation (r = 0.441, p < 0.01, n = 37). Goats in late gestation had significantly higher IGF-1 than goats in early, mid-gestation, and the last 15 days before delivery (p < 0.05), but not different from non-pregnant goats. Leptin concentration was not correlated with the day of gestation and was not different among gestation periods (Table-2).

### Relationships between dams’ IGF-1/leptin serum concentration during gestation and growth performance of kids

Thirty-four dams in this study had a mean litter size of 1.91 ± 0.12 (range 1–3), while the mean parity was 3.59 ± 0.26 (range 2–7).

The growth performance of the kids was not correlated with the dam’s IGF-1 at early, mid-, and late gestation, except for C10 at mid- (p < 0.05) (Table-3). However, all growth performances of the kids were positively correlated with the dam’s IGF-1 measured at ≤15 days before delivery. Unlike IGF-1, the dam’s leptin obtained at mid-gestation negatively correlated with the kid conformation at 10 weeks of age and ADG (Table-4).

**Table-3:** Relationship between serum IGF-1 levels in dams at different gestation periods and kid’s growth performance.

Growth performance	IGF-1 (ng/mL)			

	E (1–50 days)	M (51–100 days)	L (101–135 days)	The last 15 days (136–150 days)
W0 (kg)				
r	0.140	−0.204	0.311	0.734
p	0.591	0.448	0.209	0.00282
H0 (cm)				
r	−0.342	−0.109	0.204	0.850
p	0.179	0.687	0.418	0.000118
L0 (cm)				
r	0.146	−0.472	0.301	0.677
p	0.577	0.0649	0.226	0.00789
C0 (cm)				
r	0.00409	−0.185	0.086	0.706
p	0.988	0.493	0.736	0.00475
W10 (kg)				
r	0.00822	−0.399	-0.199	0.734
p	0.976	0.140	0.444	0.00431
H10 (cm)				
r	−0.0901	−0.188	−0.200	0.781
p	0.740	0.503	0.442	0.00162
L10 (cm)				
r	−0.157	−0.227	−0.299	0.747
p	0.560	0.416	0.243	0.00333
C10 (cm)				
r	0.140	−0.548	−0.184	0.663
p	0.604	0.0344	0.479	0.0135
ADG0–4 W (g/day)				
r	−0.414	−0.475	−0.132	0.855
p	0.0985	0.0631	0.613	0.0000968
ADG4–8 W (g/day)				
r	0.124	−0.0569	−0.253	0.543
p	0.648	0.834	0.324	0.0550

E=Early gestation, M=Mid gestation, Late=Late gestation, r=Correlation coefficient, p*=*p-value, W0=Birth weight, H0=Height at birth, L0=Length at birth, C0=Chest girth at birth, W10=Weight at 10 weeks, H10=Height at 10 weeks, L10=Length at 10 weeks, C10=Chest girth at 10 weeks, ADG0–4 W=Average daily gain between birth and 4 weeks, ADG4–8 W=Average daily gain between 4 and 8 weeks, IGF-1=Insulin-like growth factor 1

**Table-4:** Relationships between serum leptin levels in dams at different gestation periods and kid’s growth performance.

Growth performance	Leptin (ng/mL)			

	E (0–50 days)	M (51–100 days)	L (101–135 days)	The last 15 days (136–150 days)
W0 (kg)				
r	0.199	−0.439	−0.109	0.155
p	0.445	0.0888	0.668	0.690
H0 (cm)				
r	0.387	−0.490	0.179	0.429
p	0.125	0.0539	0.477	0.249
L0 (cm)				
r	0.183	−0.116	−0.016	0.150
p	0.481	0.668	0.951	0.700
C0 (cm)				
r	0.262	−0.296	0.159	0.456
p	0.310	0.266	0.530	0.218
W10 (kg)				
r	−0.191	−0.664	0.346	0.290
p	0.478	0.0069	0.174	0.485
H10 (cm)				
r	−0.235	−0.682	0.254	0.504
p	0.380	0.00511	0.325	0.203
L10 (cm)				
r	0.00535	−0.664	0.355	0.454
p	0.984	0.00696	0.162	0.258
C10 (cm)				
r	−0.155	−0.468	0.292	0.320
p	0.568	0.0782	0.255	0.439
ADG0–4 W (g/day)				
r	−0.252	−0.531	0.251	0.321
p	0.329	0.0341	0.332	0.400
ADG4–8 W (g/day)				
r	−0.663	−0.611	0.404	0.331
p	0.553	0.0118	0.108	0.423

E=Early gestation, M=Mid gestation, Late=Late gestation, r=Correlation coefficient, p*=*p-value, W0=Birth weight, H0=Height at birth, L0=Length, at birth, C0=Chest girth at birth, W10=Weight at 10 weeks, H10=Height at 10 weeks, L10=Length at 10 weeks, C10=Chest girth at 10 weeks, ADG0–4 W=Average daily gain between birth and 4 weeks, ADG4–8 W=Average daily gain between 4 and 8 weeks, IGF-1=Insulin-like growth factor 1

### Growth performance of kids born from dams with low and high concentrations of IGF-1 and leptin measured ≤15 days before delivery

The growth performances of kids born from dams with high and low concentrations of IGF-1/leptin measured ≤15 days before delivery are shown in Table-5. Kids born from dams with high serum IGF-1 levels had significantly higher H0 and ADG0–4 W than those born from dams with low serum IGF-1 (p < 0.05). Kids born from dams with high leptin levels had significantly higher C0, L10, and C10 and tended to have higher H0, W10, ADG0–4 W, and ADG4–8 W than those born from dams with low leptin levels (Table-5).

**Table-5:** Growth performance of kids born from dams with low and high concentrations of IGF-1 and leptin measured during the last 15 days before delivery.

Growth performance	IGF-1 (ng/mL)		p-value	Leptin (ng/mL)		p-value
	
	High	Low		High	Low	
W0 (kg)	1.62 ± 0.08	1.50 ± 0.13	0.439	1.64 ± 0.18	1.48 ± 0.13	0.514
H0 (cm)	31.5 ± 0.5	29.5 ± 0.7	0.038	31.6 ± 0.5	29.9 ± 0.4	0.065
L0 (cm)	28.5 ± 1.1	28.2 ± 1.8	0.913	28.3 ± 2.6	27.7 ± 1.9	0.857
C0 (cm)	27.8 ± 0.7	25.7 ± 1.0	0.112	27.6 ± 0.4	26.2 ± 0.3	0.047
W10 (kg)	8.26 ± 0.53	7.04 ± 0.88	0.254	9.44 ± 0.65	7.11 ± 0.45	0.062
H10 (cm)	47.0 ± 1.0	43.3 ± 1.7	0.111	48.9 ± 2.6	44.2 ± 1.8	0.232
L10 (cm)	50.1 ± 2.0	45.4 ± 3.3	0.271	54.7 ± 1.5	45.7 ± 1.0	0.016
C10 (cm)	45.5 ± 1.0	44.2 ± 1.6	0.517	47.8 ± 0.9	44.1 ± 0.6	0.045
ADG0–4 W (g/day)	102.0 ± 6.6	74.7 ± 10.0	0.046	111.6 ± 12.2	78.3 ± 8.3	0.096
ADG4–8 W (g/day)	82.3 ± 8.7	66.9 ± 14.2	0.377	101.9 ± 10.6	67.4 ± 7.4	0.078

Data are presented as mean ± standard error of mean, p*=*p-value, W0=Birth weight, H0=Height at birth, L0=Length, at birth, C0=Chest girth at birth, W10=Weight at 10 weeks, H10=Height at 10 weeks, L10=Length at 10 weeks, C10=Chest girth at 10 weeks, ADG0–4 W=Average daily gain between birth and 4 weeks, ADG4–8 W=Average daily gain between 4 and 8 weeks. IGF-1; high and low using mean as a cutoff (IGF-1; high: >231.6 ng/mL and low: ≤231.6 ng/mL, or leptin; high: >3.0 mg/dL and low: ≤3.0 mg/dL). IGF-1=Insulin-like growth factor 1

## Discussion

### Serum IGF-1 and leptin levels in dams and prepubertal goats

The average growth rate of goats in this study is comparable to that in a previous study by Nuntapaitoon *et al*. [9] of the same breed. IGF-1 and leptin concentrations in dams were higher than in prepubertal goats. The concentration of IGF-1 was comparable to a previous study by Devrim *et al*. [10] in Honamlı and native hair goats in Turkey, in which it increased with age, and the concentration at 4 months was lower than those measured at 8 and 12 months. However, in growing Angola goat kids, circulating IGF-1 decreased from 12 to 22 weeks of age [11]. Two studies in goats showed that plasma IGF-1 levels in kids after birth were elevated until approximately 4 months of age and declined without significance thereafter [5, 12]. They concluded that the changes in plasma IGF-1 concentration may be due to temperature changes that increased with increased THI. However, the effect of age on IGF-1 expression could not be ruled out.

Lower leptin in prepubertal goats compared with dams is similar to a previous study by Vitali *et al*. [13] that showed that increased plasma leptin concentration was found during the onset of puberty in goats. Leptin levels in goat kids at parturition were 50% lower than those found in maternal plasma and increased significantly with age [7]. A report of higher plasma leptin in goats at 8 and 12 months than at 4 months of age was found [10], although the concentration of leptin was four times higher than that in the present study, which may be due to the measurement method. A previous report by Karaayvaz *et al*. [14] of lower serum leptin in Honamlı goats (3.38 ± 0.76 ng/mL) aged between 24 and 72 months than in Hair goats (5.48 ± 0.92 ng/mL) aged between 96 and 120 months was also found, which may be due to age difference. Moreover, leptin concentration was correlated with age in ewes [15].

### Serum IGF-1 and leptin concentrations in dams with different age and gestation period

The concentrations of both IGF-1 and leptin in dams were not different among age groups. IGF-1 increased during gestation, and the highest was found during late gestation. IGF-1 was dropped 15 days before delivery. A previous study by Hashizume *et al*. [16] showed that plasma IGF-1 concentrations during late gestation were higher than the post-parturition concentration, which may result from either elevated placental lactogen or the GH variant at late gestation, and IGF-1 gradually declined 6 weeks before parturition. Similar results in the transition period have been observed in Angora does [17]. However, in growing kids, IGF-1 levels were high from 1 week after birth and declined thereafter until 12 weeks of age. It is possible that a decrease in maternal IGF-1 levels before parturition and a high IGF-1 level in the 1^st^ week in kids involve mammary production and secretion of IGF-1 in colostrum. Nevertheless, high IGF-1 levels in the fetus in response to high concentrations of growth hormone during gestation remain to be clarified.

The concentration was variable for leptin, with no differences found among reproductive cycles. The concentration during gestation tended to be higher than that in non-pregnant goats in the dry period (125.5 ± 27.3 days after delivery). Although plasma leptin concentrations increased during pregnancy due to production from the placenta [18], other factors may be involved, such as fat utilization, intake, and receptor responsiveness under different physiological conditions, lactation with or without pregnancy. A previous study by Bonnet *et al*. [19] showed a sharp increase in leptin concentration in non-pregnant goats but not in pregnant goats in late lactation drying off. It was reported to be high during pregnancy and declined to a nadir at parturition in cows [20]. Leptin concentrations are greatest during the dry period, decrease around the parturition period, and remain low in early lactation compared with values before calving. Reducing leptin during parturition and early lactation contributes to the adaptive mechanism and stimulates hyperphagia [6].

Rather than the reproductive cycle, other factors can affect leptin concentration, including litter size and environmental conditions. Nulliparous goats had higher leptin concentrations than primiparous goats in the 1^st^ month of pregnancy [19]. In addition, higher leptin levels in goats during chronic heat exposure were found in Salem Black goats but not in Osmanabadi goats [21]. In this study, all BB goats were raised at the same environmental temperature, and blood collection for leptin measurements was performed on the same day.

### Relationships between serum IGF-1/leptin in dam and kid’s growth performance

IGF-1 levels in dams with early, mid, and late gestation did not correlate with kid growth performance. However, growth performance at birth, week 10, and ADG 1-month postpartum was highly associated with the dam’s IGF-1 level measured ≤15 days before delivery. The effect of IGF-1 at birth may indirectly affect maternal metabolism or placental development because IGF-1 was reported not to cross the placenta in cows [22]. Infusion intravenously with IGF-1 for 1 week into late gestation fetal sheep showed increased weight of some organs, whereas umbilical amino acid uptake and plasma amino acid of fetus reduced [23]. It is believed that IGF-1 causes efficient nutrient usage rather than increased placental blood flow or nutrient transfer to the fetus. The role of hormones in nutrient transport across the placenta was reviewed, while IGF-1 regulates glucose uptake by stimulating GLUT1 receptor expression in the placenta [24].

Kids born from dams with higher serum IGF-1 levels during the last 15 days before delivery had higher ADG in the 1^st^ month postpartum. This may be a passive transfer of maternal IGF-1 via colostrum. A very high concentration of IGF-1 in the colostrum of goats was found, and it was reduced tremendously 3 days after delivery [2], whereas the colostrum IGF-1 concentration was associated with kid growth at 1 month postpartum [4]. IGF-1 from colostrum enhances gastrointestinal tract development in kids and promotes high immunity [25, 26].

The leptin concentration in dams during mid-gestation negatively correlated with the growth of kids at 1–2 months postpartum. The results could not be explained when leptin concentration correlated positively with the pregnancy term being advanced. Kids from dams with high leptin levels tended to perform better at 10 weeks and higher ADG at 4- and 8-week postpartum. The role of leptin in utero and the passive transfer of leptin is unclear. In humans, leptin can promote fetal development in the placenta [27]. However, ruminants produce negligible expression of placental leptin [18]. Moreover, leptin concentrations in arterial and milk venous plasma were highest during late gestation, decreased after parturition, and remained low postpartum compared with prepartum, whereas leptin in milk increased sharply and reached a peak 2-day postpartum. Intake of colostrum after parturition had no effect on plasma leptin levels in kids 6 h after that. In addition, plasma leptin levels in kids were independent of milk leptin and plasma maternal leptin [7]. A decline in plasma leptin concentration in suckling kids from 3 to 5 weeks of lactation while kids consumed higher milk intake was demonstrated in goats [28]. Thus, the role of passive transfer of maternal plasma leptin on the growth and development of kids is negligible.

Leptin affects the growth rate of kids, as has been shown by many studies. In cows, leptin mRNA and protein expression in the gastrointestinal tract of calves, especially at the abomasum and jejunum, was higher than in mature cows, which may play an important role during the suckling period in ruminants [8]. The leptin gene has been studied extensively and reported to be related to growth, carcass, milk, and reproductive traits in cattle [29]. A single nucleotide polymorphism of the leptin gene was associated with growth traits in Jamunapari goats [30]. Moreover, leptin receptor polymorphisms are associated with litter size in BB goats [31].

Leptin concentration during the last 2 weeks of delivery may reflect the nutritional status of dams, which is related to body condition score, body fat storage, and nutrient use. In sheep, plasma leptin concentration was positively correlated with the body condition score [32]. Underfed ewe exhibited a reduction in plasma leptin concentration. The relationship between leptin and body condition score was also demonstrated in goats [33]. Therefore, leptin levels in dams at the late gestation period may reflect dams’ nutritional status and energy utilization. Plasma leptin concentrations were lower in cows with a negative energy balance during lactation with high milk yield, low feed intake, and low live weight [20].

### Limitation

Due to technical difficulties in the field, blood collection of dams was performed on the same day with different gestation periods. It will be interesting to see the changes in circulating IGF-1 and leptin concentrations that were obtained from the same dams during different gestation periods.

## Conclusion

Serum IGF-1 concentration in immature goats was lower than that in mature goats. Mean serum IGF-1 concentrations were higher in the dam sample later during pregnancy. The IGF-1 concentration collected from dam ≤15 days before delivery correlated positively with kid growth traits, while ADG0–4 W was higher in kids from dams with higher IGF-1 than those from dams with low IGF-1. Moreover, kids from dams with high leptin also tended to grow more at 4–8 weeks postpartum. Thus, IGF-1 and leptin concentrations in dams measured ≤15 days before delivery were associated with kid growth performance.

## Authors’ Contributions

CB, SC, and MN: Data curation. CB and MN: Formal analysis. CB and SC: Methodology. CB: Project administration; CB: Original draft. CB and MN: Supervision and editing. All authors have read, reviewed, and approved the final manuscript.

## References

[1] Hossain M.E (2021). Performance of Black Bengal goat:A 50-year review. Trop. Anim. Health Prod.

[2] Buranakarl C, Thammacharoen S, Semsirmboon S, Sutayahtam S, Nuntapaitoon M, Dissayabutra T, Kato K (2021). Effects of litter size and parity number on mammary secretions including, insulin-like growth factor, immunoglobulin G and vitamin A of Black Bengal, Saanen and their crossbred goats in Thailand. Vet. Sci.

[3] Cardoso C.L, King A, Chapwanya A, Esposit G (2021). Ante-natal and post-natal influences on neonatal immunity, growth and puberty of calves - a review. Animals (*Basel*).

[4] Buranakarl C, Thammacharoen S, Semsirmboon S, Sutayahtam S, Nuntapaitoon M, Kato K (2022). Impact of insulin-like growth factor 1, immunoglobulin G and vitamin A in colostrum on growth of newborn Black Bengal goats and its crossbred. J. Anim. Physiol. Anim. Nutr. (*Berl*).

[5] Pehlivan E (2019). Relationship between insulin-like growth factor-1 (IGF-1) concentrations and body trait measurements and climatic factors in prepubertal goat kids. Arch. Anim. Breed.

[6] Häussler S, Sadri H, Ghaffari M.H, Sauerwei H (2022). Symposium review:Adipose tissue endocrinology in the periparturient period of dairy cows. J. Dairy Sci.

[7] Rasmussen A.N, Nielsen M.O, Tauson A.H, Offenberg H, Thomsen P.D, Blach D (2008). Mammary gland leptin in relation to lactogenesis in the periparturient dairy goat. Small Rumin. Res.

[8] Hayashi H, Yamakado M, Yamaguchi M, Kozaka T (2020). Leptin and ghrelin expressions in the gastrointestinal tracts of calves and cows. J. Vet. Med. Sci.

[9] Nuntapaitoon M, Buranakarl C, Thammacharoen S, Kato K (2021). Growth performance of Black Bengal, Saanen, and their crossbred F1 as affected by sex, litter size, and season of kidding. Anim. Sci. J.

[10] Devrim A.K, Elmaz O, Mamak N, Sudagida M (2015). Levels of hormone and cytokines associated with growth in Honamlıand native hair goats. Pol. J. Vet. Sci.

[11] Acuti G, Todini L, Malfatti A, Antonini M, Barbato O, Trabalza-Marinucc M (2009). Effect of field bean (*Vicia faba* L. var. minor) dietary supplementation on plasma thyroid hormones, insulin, insulin-like growth factor-1 concentrations and mohair characteristics in growth Angora goat kids. J. Anim. Physiol. Anim. Nutr.

[12] Deori S, Abedin S.N, Chakravaerty H, Das S, Katiyar R, Dole S (2023). Exploring the link between insulin-like growth factor-1 (IGF-1) and body trait measurements in prepubertal goat kids in a humid subtropical climate. Indian J. Anim. Res.

[13] Vitali A, Magistrelli D, Azevedo J, Bernabucci U, Ronchi B, Ros F (2005). Leptin and puberty in goat. Ital. J. Anim. Sci.

[14] Karaayvaz B.K, Kıyıcı R, Öztürk Y, Bağcı I, Gürsoy T, Kahraman D, Akkan H.A, Mamak N, Taşal I, Karac M (2022). Determination of serum leptin levels in cattle, sheep, goats and buffaloes in Burdur Province in Türkiye by ELISA method. Cumhuriyet Univ. Sağlık Bilim. Enst. Derg.

[15] Kuznicka E, Kunowska-Slosarz M, Gabryszu M (2020). The ovulation rate, plasma leptin concentration, and litter size of a local ewe breed kept in a barn versus those kept under an overhead shelter. Agriculture.

[16] Hashizume T, Takahashi Y, Numata M, Sasaki K, Ueno K, Ohtsuki K, Kawai M, Ishi A (1999). Plasma profiles of growth hormone, prolactin and insulin-like growth factor-1 during gestation, lactation and the neonatal period in goats. J. Reprod. Dev.

[17] Todini L, Galbraith H, Malfatti A, Gabriele A, Acuti G, Barbato O, Antonini. M, Daniela B, Trabalza-Marinucc M (2022). Response to dietary supplementation of field bean (*Vicia faba* L. var. minor) in production indices, mohair growth and hormonal parameters in transition Angora goats. Ital. J. Anim. Sci.

[18] Agarwal R, Rout P.K, Sing S.K (2009). Leptin:A biomolecule for enhancing livestock productivity. Indian J. Biotechnol.

[19] Bonnet M, Delavaud C, Rouel J, Chilliar Y (2005). Pregnancy increases plasma leptin in nulliparous but not primiparous goats while lactation depressed it. Domest. Anim. Endocrinol.

[20] Liefers S.C, Veekamp R.F, te Pas M.F.W, Delavaud C, Chilliard Y, van der Lend T (2003). Leptin concentration in relation to energy balance, milk yield, intake, live weight, and estrus in dairy cows. J. Dairy Sci.

[21] Archana P.R, Sejian V, Ruban W, Bagath M, Krishnan G, Aleena J, Manjunathareddy G.B, Beena V, Bhatt R (2018). Comparative assessment of heat stress induced changes in carcass traits, plasma leptin profile and skeletal muscle myostatin and HSP70 gene expression patterns between indigenous Osmanabadi and Salem Black goat breeds. Meat Sci.

[22] Davenport M.L, D'Ercole A.J, Underwoo L.E (1990). Effect of maternal fasting on fetal growth, serum insulin-like growth factors (IGFs), and tissue IGF messenger ribonucleic acids. Endocrinology.

[23] Stremming J, Heard S, White A, Chang E.I, Shaw S.C, Wesolowski S.R, Jonker S.S, Rozance P.J, Brow L.D (2021). IGF-1 infusion to fetal sheep increases organ growth but not by stimulating nutrient transfer to the fetus. Am. J. Physiol. Endocrinol. Metab.

[24] Toschi P, Baratt M (2021). Ruminant placental adaptation in early maternal undernutrition:An overview. Front. Vet. Sci.

[25] Yang M, Zou Y, Wu Z.H, Li S.L, Ca Z.J (2015). Colostrum quality affects immune system establishment and intestinal development of neonatal calves. J. Dairy Sci.

[26] Hammon H.M, Liermann W, Frieten D, Koc C (2020). Review:Importance of colostrum supply and milk feeding intensity on gastrointestinal and systemic development in calves. Animal.

[27] Bodner J, Ebenbichler C.F, Wolf H.J, Müller-Holzner E, Stanzi U, Gander R, Huter O, Patsc J.R (1999). Leptin receptor in human term placenta:In situ hybridization and immunohistochemical localization. Placenta,.

[28] Magistrelli D, Polo Dimel G, Ros F (2008). Leptin, insulin and ghrelin levels in goat milk and in plasma of suckling kids. Small Rumin. Res.

[29] Nugroho T, Widi T.S.M, Maharan D (2022). The Potency of Leptin Gene as a Selective Marker of Economic Traits for Madura Cattle:Preliminary Study. In:Proceedings of the 9^th^ International Seminar on Tropical Animal Production (ISTAP 2021):Advances in Biological Sciences Research, Yogyakarta, Indonesia, Atlantis Press International B.V.

[30] Dige M.S, Kaushik R, Mishra C, Verma M, Pawaiya R, Bhusan S, Rou P.K (2021). Association of single nucleotide polymorphism in leptin gene with growth traits of Jamunapari goat. Indian J. Anim. Sci.

[31] Alim M.A, Hossain M.M.K, Nusrat J, Rubaya Salimulla M, Shu-Hon Z, Alam J (2019). Genetic effects of leptin receptor (LEPR) polymorphism on litter size in a Black Bengal goat population. Anim. Biol.

[32] Delavaud C, Bocquier F, Chilliard Y, Keisler D.H, Gertler A, Kan G (2000). Plasma leptin determination in ruminant:Effect of nutritional status and body fatness on plasma leptin concentration assessed by a specific RIA in sheep. J. Endocrinol.

[33] Gamez-Vazquez H.G, Rosales-Nieto C.A, Banuelos-Valenzuela R, Urrutia-Morales J, Olivia-Diaz Gomez M, Silva-Ramos J.M, Meza-Herrer C.A (2008). Body condition score positively influence plasma leptin concentrations in Criollo goats. J. Anim. Vet. Adv.

